# Rapid Administration of Antibiotics for Reducing Fever Days in Patients Receiving Hematopoietic Stem Cell Transplantation

**DOI:** 10.3390/medicina58091157

**Published:** 2022-08-25

**Authors:** Ming-Yang Lee, Yu-Ju Chang, Yin-Che Lu, Chin-Ho Kuo, Ya-Hui Kuo, Shu-Chien Tzeng, Gwo-Jong Hsu

**Affiliations:** 1 Division of Hemato-Oncology, Department of Internal Medicine, Ditmanson Medical Foundation Chiayi Christian Hospital, Chiayi 60002, Taiwan; 2 Min-Hwei Junior College of Health Care Management, Tainan 73658, Taiwan; 3 Department of Nursing Care, Ditmanson Medical Foundation Chiayi Christian Hospital, Chiayi 60002, Taiwan; 4 Division of Infection, Department of Internal Medicine, Ditmanson Medical Foundation Chiayi Christian Hospital, Chiayi 60002, Taiwan

**Keywords:** hematopoietic stem cell transplantation, neutropenia fever, antibiotics injection

## Abstract

*Background and Objectives*: Neutropenic fever (NF) is a major cause of mortality and morbidity in patients undergoing hematopoietic stem cell transplantation (HSCT). To date, no study has discussed the relationship of fever days in HSCT with the time between recording the fever and administering antibiotics. This study aimed to examine the association between fever days in HSCT and the time interval between recording the fever and intravenous (IV) antibiotics to the febrile neutropenia patient. *Materials and Methods*: A total of 22 patients who developed NF after HSCT in one hospital were analyzed. Patients who received IV antibiotics injection within 30 min were categorized in group A and those who received the injection after 30 min were categorized in group B. Fever was defined by an attack with an oral temperature of 38.3 °C. Patients’ characteristics and possible risk factors were recorded and analyzed. *Results*: Groups A and B had 14 and 8 patients, respectively. Patient characteristics, including age, diagnosis, sex, and antibiotics level, were similar between the two groups. The median duration of fever days was 1.5 (range, 1–5) in group A and 6.5 (range, 1–14) in group B (*p* = 0.003). Multivariant analysis of possible independent impact factors of “fever days in HSCT” was performed. The odds ratio of “antibiotics given time” was 4.00 (95% confidence interval [CI] = 2.26 to 7.22, *p* = 0.001). The “antibiotics level” did not affect the NF period (odds ratio = −0.80, 95% CI = −2.40 to 1.07, *p* = 0.453). *Conclusions*: Rapid IV administration of antibiotics (<30 min after fever attack) can reduce the fever days in patients undergoing HSCT.

## 1. Introduction

High-dose chemotherapy with stem-cell rescue may effectively eliminate residual disease, reduce relapse risk, and prolong disease-free survival in hematological disorders, such as malignant lymphoma, leukemia, and multiple myeloma [1,2,3,4]. However, fever during neutropenia (neutropenic fever (NF)) is almost universally present in complications of autologous and allogeneic hematopoietic stem cell transplantation (HSCT) recipients [5]. Fever may be the only manifestation of infection, because NF patients are unable to initiate an appropriate immune response.

Although the application of new broad-spectrum antibiotics (BSA) and the granulocyte colony-stimulating factor (G-CSF) have reduced the fever days of patients receiving HSCT, infection is a major complication of HSCT [6,7,8,9,10,11,12]. Several practice guidelines have suggested that isolation protection; central vein catheter care; prophylactic antibacterial, antifungal, and antiagents use; oral hygiene care; and G-CSF [13,14] are effective at reducing the incidence of NF [4,8,13,14,15,16,17,18,19,20,21]. Thus, effective prevention of specific infections has allowed for the use of increasingly effective conditional therapies, thereby prolonging survival in HSCT recipients [22].

When a fever episode occurs, the administration of empirical broad-spectrum antibiotics as soon as possible is recommended [13,14,15,16,17,18,19,20,21]. The Infectious Disease Society of America and the National Comprehensive Cancer Network recommend time to antibiotic for NF patients of 60 to 120 min [23]. However, no study has discussed the relationship of fever days in HSCT with the time between recording the fever and administering antibiotics. Therefore, in this study, we analyzed the association between fever days in HSCT and the time interval between recording the fever and intravenous (IV) antibiotics to the NF patient.

## 2. Materials and Methods

### 2.1. Patients

Adult patients with hematological disorder who received HSCT were recruited for the study from one institute. This retrospective study was approved by the Institutional Review Board of Chiayi Christian Hospital (approval no. CYCH-IRB-104019). All patients received HSCT in the same laminar airflow room, and their body temperatures were recorded every 4 h if their body temperature was <37 °C, and every 1 h if body temperature was >37 °C. Patient characteristics and possible risk factors influencing fever days in HSCT, including age, antibiotics level, and antibiotics given time, were recorded through chart review. The posttransplant follow-up was continued until 6 months after transplant.

### 2.2. Conditioning Treatments

The conditioning regimen used for malignant lymphoma in 15 patients was BEAC (i.e., bis-chloroethylnitrosourean (BCNU) 300 mg/m^2^/day on day 7, etoposide 100 mg/m^2^/day from day 6 to day 3, cytarabine 100 mg/m^2^/day from day 6 to day 3, and cyclophosphamide 35 mg/kg/day from day 6 to day 3) [1]. The conditioning regimen for three patients with acute myeloid leukemia and two patients with acute lymphoid leukemia patients was BuCy (busulfan (1 mg/kg body weight) orally every 6 h for 16 doses from day 7 to day 4, followed by cyclophosphamide (60 mg/kg/day IV) after 1 h on day 3 and day 2) [2]. The conditioning regimen for three patients with severe aplastic anemia and one patient with multiple myeloma was high-dose cyclophosphamide (50 mg/kg/day IV) after 1 h from day 5 to day 2 [3].

### 2.3. Infection Prophylaxis

All patients were managed in a laminar airflow room under standardized care procedures. Prophylaxis with oral antibiotics (trimethoprim-sulfamethoxazole and levofloxacin) and an antifungal agent (fluconazole) was provided from day 10 to patients transferred to the general ward. When the patient started switching to broad-spectrum antibiotics, prophylaxis oral antibiotics were discontinued at the same time, but antifungal agents continued. Cytomegalovirus prophylaxis was used in all of the patients [6,10,13]. All of the patients received G-CSF subcutaneous injection (300 mcq per day) from the day of transplantation until the patient’s absolute neutrophil counts were >3000/cumm.

### 2.4. Definition of Fever Days in HSCT

Neutropenia was defined as an absolute neutrophil count of less than 500/µL. Fever was defined by an attack with an oral temperature of 38.3 °C. “Fever days in HSCT” was defined as the number of days on which the HSCT patient had one or more medical records of oral temperature >38.3 °C. Subsided fever was defined as a patient oral temperature of <38.3 °C for 24 h. Acute graft-versus-host disease was excluded based on clinical expressions.

### 2.5. Definition of “Antibiotics Given Time”

The time at which the oral temperature of the HSCT patient was >38.3 °C was defined as “time point A”. The time at which the patient was injected with IV antibiotics was defined as “time point B”. The “antibiotics given time” was defined as the time interval between “time point A” and “time point B”. In this study, patients in Group A received IV antibiotics within 30 min and those in Group B received IV antibiotics >30 min after fever was noted.

### 2.6. Definition of “Antibiotics Level”

Antibiotics that are highly potent against broad-strain aerobic Gram-negative bacilli, including *Pseudomonas aeruginosa*, such as carbapenems and various antipseudomonal penicillins, and those with a potent activity against broad-strain aerobic Gram-positive bacilli, including methicillin-resistant *Staphylococcus aureus* (MRSA), such as vancomycin as well as anti-fungal treatments, are defined as level II antibiotics. Other antibiotics that have minimal activity against *P. aeruginosa* or MRSA are defined as level I antibiotics.

### 2.7. Statistical Analysis

The SPSS software (Version 12.0; SPSS, Inc., Chicago, IL, USA) was used for the statistical analysis. The chi-square or Fisher exact test was used for testing the dichotomous variables. The factors that were significant or that showed a trend toward significance based on univariate analysis were used for multiple regression analysis. The level of statistical significance was set at <0.05.

## 3. Results

In total, 24 adult patients with hematological disorder who received HSCT were enrolled. The vital sign check-up, infection prophylaxis, graft-versus-host disease (GVHD), and seizure prophylaxis were performed for all patients at the same time schedules. In this study, 22 adult patients had NF. Of the patients with NF, 14 received IV antibiotics within 30 min (group A) of fever occurrence and the remaining eight received IV antibiotics >30 min after fever occurrence (group B). In group B, five patients received IV antibiotics for 30–60 min (three received class I antibiotics and two received class II therapy); the other three patients received IV antibiotics >60 min (one received class I antibiotics treatment and two received level II treatment). Other hematological disorders included three with severe aplastic anemia, three with acute myeloid leukemia, two with acute lymphoblastic leukemia, and one with multiple myeloma. Acute GVHD, including skin presentation (dermatitis and blisters), abdominal upset (severe diarrhea and nausea), and hepatitis without other etiologies were closely followed up clinically. The patient characteristics, including age, underlying diagnosis, sex, and antibiotics level, were similar between the two groups (Table 1).

**Figure 1:**
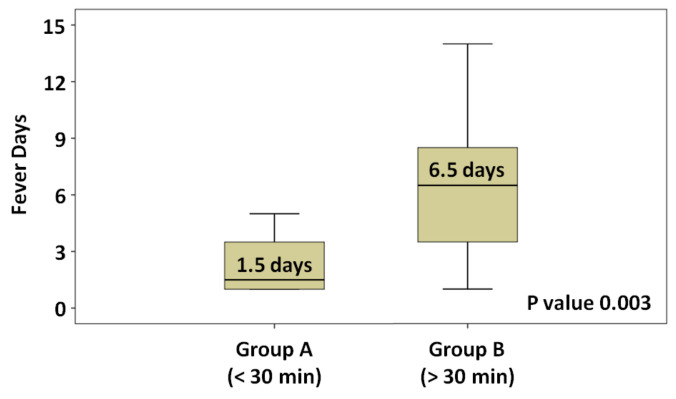
Median duration of fever days.

The median duration of fever days was 1.5 (range, 1–5) in group A and 6.5 (range, 1–14) in group B (*p* = 0.003) (Figure 1). Multivariant analysis of possible independent impact factors for “fever days in HSCT” were examined in two risk variants, namely “antibiotics given time” and “antibiotics level”. The odds ratio (OR) for antibiotics given time (group B vs. group A) was 4.00 (95% confidence interval [CI] = 2.26 to 7.22, *p* = 0.001). The OR for antibiotics level (first-line antibiotics vs. second-line antibiotics) was −0.80 (95% CI = −2.40 to 1.07, *p* = 0.453) (Table 2).

## 4. Discussion

Bacterial infection is a major concern among patients receiving HSCT and may lead to high mortality (1.5–28%) during neutropenia status [9,11,12]. Risk factors associated with bacterial infection in these patients are HSCT type, granulocytopenia, mucositis, presence of central venous catheter, presence of graft-versus-host disease, and contamination of hematopoietic stem cell collection [9,18]. Infections remain the leading cause of hematopoietic stem cell transplantation morbidity, mortality, length of hospital stay, and healthcare costs [24]. Antibiotics prophylaxis is suggested routinely. However, even after nursing care and infection prophylaxis are integrated, the incidence of febrile neutropenia is high, and empirical antibiotics administration is crucial as fever episodes occur during HSCT [7,9,13,14]. In most guidelines regarding the management of febrile neutropenia, the focus is on the choice of antibiotics and not on how soon empirical antibiotics must be administered [6,7,8,13,14,15,16,17,18,19,20,21]. In our results, we did not observe a statistical difference between different types of antibiotics. This may be due to the small sample size of the study.

Patients undergoing HSCT typically develop a diverse range of symptoms during bone marrow recoveries, such as fever, which is thought to reflect a systemic inflammatory state corresponding to immune cell reconstitution, associated with cytokine release and capillary leakage. In adult patients who received HSCT, it was found that the length of hospital stay was directly related to the frequency of fever (*p* < 0.0001), and the incidence of fever in autologous stem cell transplantation was significantly higher than that in allogeneic transplantation (*p* < 0.05) [25]. Patients aged 60 years or older are known to be more likely to develop peri-engraftment fever than younger patients (1.833, 1.045–3.214, *p* = 0.034) [26].

Fever is the most important sign of infection during the early post-transplantation before engraftment. Fever days could be associated with the duration of neutropenia, timing of fever relative to engraftment/HSCT, the timing of graft engraftment after HSCT, and the source of graft (peripheral blood, bone marrow, or cord blood), especially in allogeneic HSCT. Allogeneic HSCT patients had a longer duration of neutropenia than autologous patients (15.6 vs. 9.2 days, *p* < 0.001) [27]. Additionally, some practice guidelines have indicated that IV antibiotics must be administered as soon as possible based on general practice experience and not according to scientific statistical analysis [6,7,8,13,14,15,16,17,18,19,20,21,28]. Furthermore, knowing how fast is acceptable and how slow is critical, and remains unclear. According to a study by Jeddi et al. [28], the first 6 h is the ideal time to administer IV BSA and any delay may increase mortality. In our retrospective analysis, we observed that administering antibiotics within 30 min can reduce febrile days compared with administering antibiotics >30 min after fever attack (1.5 vs. 6.5 days, *p* = 0.003).

Lin et al. [29] observed that patients with severe neutropenia (absolute neutrophil count (ANC) < 100 cumm) treated in a nonintensive care unit with delayed active antimicrobial therapy showed a high mortality (OR = 18.0; 95% CI = 2.84 to 114.5; *p* < 0.01). However, patients with severe neutropenia but treated in an intensive care unit with delayed active antimicrobial therapy show a trend of increased mortality (OR = 5.56; 95% CI = 0.85 to 36.3; *p* = 0.07). In this study, one case of delayed active IV antimicrobial therapy involved initiation after 24 h of index blood withdrawal. However, in our study, we ensured adequate IV antibiotics administration within 30 min from fever occurrence. This treatment regimen reduced the fever days in intensive care unit statistically and may improve survival outcomes.

This study had some limitations. First of all, it is a single-center retrospective cohort analysis; its applicability to large programs across the country is not yet known. Second, the sample size in this study was relatively small, making it more difficult to distinguish the real effect and random variation. Lastly, in most hospital administration policies in Taiwan, IV antibiotics are stored in the central pharmacy, which may lead to time loss during the transit of medications to the HSCT room. These security barriers may delay the timely administration of IV antibiotics after fever occurrence.

## 5. Conclusions

Fever during neutropenia is almost universal after an HSCT. In this preliminary result, we found that HSCT patients needed to be given IV antibiotics <30 min after fever occurrence. This procedure can reduce the fever days in HSCT. A large sample size is needed to confirm its role in reduced mortality. For patient safety, HSCT management education should emphasize the importance of administering antibiotics to patients with neutropenic fever within 30 min of fever occurrence.

## Figures and Tables

**Table 1:** Patient characteristics and relevance of antibiotics given time and clinical variables for reducing fever days in HSCT.

	Group A (<30 min)	Group B (>30 min)	*p* Value
Number	14	8	
Median Age (range) (years)	48 (27–63)	45 (18–69)	0.661
Gender (male/female)	10/4	4/4	0.362
Diagnosis			0.818
Malignant lymphoma	8 (57.1%)	5 (62.5%)	
Other hematological disorders	6 (42.9%)	3 (37.5%)	
HSCT type			0.343
Autologous HSCT	10	4	
Allogeneic HSCT	4	4	
Antibiotics level (I/II)	5/9	4/4	0.662
Death within 30 days after HSCT	0 (0%)	1 (14.3%)	0.389

Antibiotic level I, minimal activity against *P. aeruginosa* or MRSA. Antibiotics level II, potent activity against broad strain. Abbreviations: HSCT, hematopoietic stem cell transplantation.

**Table 2:** Effects of possible risk factors on fever days in patients receiving HSCT based on multiple regression analysis.

	Relative risk of Fever Days in HSCT (95% Confidence Interval)			*p* Value
Antibiotics Given Time (>30 min vs. <30 min)	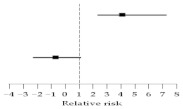	4.00	(2.26 ~ 7.22)	0.001
Antibiotics Level (I vs. II)		−0.80	(−2.40 ~ 1.07)	0.453

Abbreviations: HSCT, hematopoietic stem cell transplantation.

## Data Availability

The data presented in this study are available upon request from the corresponding author.
